# Electrical Therapy of Heart Failure

**DOI:** 10.19102/icrm.2017.081207

**Published:** 2017-12-15

**Authors:** Imran Niazi

**Affiliations:** ^1^Aurora Cardiovascular Services, Aurora Sinai/Aurora St. Luke’s Medical Centers, University of Wisconsin School of Medicine and Public Health, Milwaukee, WI, USA

**Keywords:** His bundle pacing, left bundle branch block, multipoint pacing

This year brought increasing interest in more physiologic forms of pacing for the prevention and treatment of heart failure (HF).

Permanent His bundle pacing, with depolarization of the ventricles via the intrinsic His-Purkinje system (HPS), is arguably the most physiologic form of ventricular pacing. Its practical application in narrow QRS patients was demonstrated by Deshmukh et al. in 2000.^1^ More recently, Lustgarten and colleagues showed that His-bundle pacing (HBP) was able to narrow the QRS complex in patients with left bundle branch block (BBB) to a variable extent in most patients.^2^ Barba-Pichardo et al. demonstrated similar narrowing in 81% of 16 patients.^3^ Permanent HBP was possible in 12 of these individuals with an improvement in clinical parameters and left ventricular ejection fraction (LVEF).

In another example, Ajijola et al. built upon this by reporting on a two-center pilot study of permanent HBP in a cardiac resynchronization therapy (CRT) population, published in *Heart Rhythm* this year.^4^ Permanent HBP with narrowing of the baseline QRS was possible in 16 of the 21 patients involved, with the mean QRS shrinking from 181ms ± 23 ms to 129 ms ± 13 ms. Normalization of the QRS was only possible in one patient; yet, the mean LVEF demonstrated an improvement from 27% to 41%, and New York Heart Association functional class recovered from 111 to 11. At one year, thresholds remained acceptable and all leads were stable. Interestingly, four of these 16 patients had right BBB, while the rest had left BBB.

One question to ask is, how does HBP resolve left BBB? Narula and others demonstrated this phenomenon in left BBB patients during distal but not proximal HBP.^5,6^ Presumably, longitudinal stratification of the fibers in the His bundle, coupled with intra-His block, was resolved by distal HBP. This appears to hold true even for HF patients with severely diseased ventricles in whom it may appear counterintuitive. In 27 HF patients with left BBB undergoing CRT, we were able to demonstrate significant narrowing of the QRS complex along with a shortening in intra-left ventricular conduction in 24 patients during the use of temporary HBP. Of these, the QRS normalized in 12, with a corresponding further shortening of intra-left ventricular conduction.

So, what can we conclude from this body of work? First, permanent HBP with significant (> 20%) narrowing of the paced complex is possible in about two-thirds of patients with CRT indications and either left or right BBB. Second, thresholds and lead stability appear to be acceptable in the intermediate term, with no evidence of progression of HPS disease distal to the pacing site. Third, this therapy appears to be associated with an improvement in clinical status and reverse remodeling. Obviously, these conclusions are tentative, pending a larger, randomized study in a better-defined population. Finally, it appears that the definitions of “direct” or “selective” versus “indirect” or “nonselective” HBP need to be rethought in the CRT population. Perhaps the terms employed should be His-bundle pacing with no distal delay (HBP-ND) for “direct” or “selective” HBP with complete normalization of the QRS complex, and His-bundle pacing with distal delay (HBP-DD) in place of “indirect” or “nonselective” HBP, respectively, in patients with incomplete narrowing of the native wide QRS complex.

## Multipoint left ventricular pacing

Another approach to improving the response to CRT is the concept of multipoint pacing in the left ventricle using a single quadripolar epicardial left ventricular lead. Pacing more than one site recruits more myocardium than single-site pacing does, and it is conceivable that it increases the chance that at least one of the pacing electrodes is present at a “sweet spot” (ie, not in a zone of slow conduction or overlying scar, yet in an electrically “late” area).

This concept was tested in the Multipoint Pacing (MPP) trial, published in *JACC: Clinical Electrophysiology*.^7^ The purpose of this multicenter randomized trial was to assess the safety and efficacy of pacing two different sites using the Quartet™ quadripolar left ventricular pacing lead (Abbott Laboratories, Chicago, IL, USA). In this study, MPP was compared with single-site left ventricular pacing. This trial was not powered to show superiority but rather to demonstrate safety and equal efficacy to conventional CRT. Secondary objectives were to assess which pairs of left ventricular lead electrodes produced the best clinical response and also whether the two left ventricular poles should be paced simultaneously or at different times. Patient response was assessed using a clinical composite score, as used in recent CRT trials such as Adaptive CRT.^8^

The complex study design of the MPP trial incorporated the implantation of a CRT-defibrillator system with a quadripolar left ventricular lead in 455 patients, who then underwent three months of standard single-site CRT. Following an assessment of the response data, the involved patients were then randomized to receive CRT with MPP or standard CRT.

Response was assessed at nine months in both groups, and was statistically similar, as was the rate of adverse events.

A post-hoc analysis of the MPP group showed that the best response was obtained when both left ventricular poles were stimulated simultaneously and were widely distant from each other rather than adjacent. The clinical response rate was 87% in this group.

It was calculated that MPP reduced battery life by about one year. This reduction is probably inconsequential, however, in a patient population in which we expect 50% of greater mortality over five years. If MPP is shown to increase the degree of response to CRT or to increase the rate of response significantly, then we believe the trade-off is well worth it.

## Left ventricular endocardial pacing

Pacing the left ventricular endocardium during CRT has been shown to improve hemodynamic response, but heretofore has been done using a cumbersome implant procedure and with a relatively high risk of stroke due to the implanted left ventricular lead. New technology developed recently by EBR Systems, Inc. (Sunnyvale, CA, USA) incorporates a minuscule electrode implanted in the left ventricular endocardium percutaneously via the retrograde aortic route. This system has undergone initial evaluation in the WISE-CRT trial, with a 50% response rate in patients who failed to respond to conventional epicardial CRT, albeit with a significant complication rate, likely due to the design characteristics of the early delivery system.^9^ The pivotal trial will commence in 2018, using an updated delivery system. We will await the results with interest.
